# Developmental genetic bases behind the independent origin of the tympanic membrane in mammals and diapsids

**DOI:** 10.1038/ncomms7853

**Published:** 2015-04-22

**Authors:** Taro Kitazawa, Masaki Takechi, Tatsuya Hirasawa, Noritaka Adachi, Nicolas Narboux-Nême, Hideaki Kume, Kazuhiro Maeda, Tamami Hirai, Sachiko Miyagawa-Tomita, Yukiko Kurihara, Jiro Hitomi, Giovanni Levi, Shigeru Kuratani, Hiroki Kurihara

**Affiliations:** 1 Department of Physiological Chemistry and Metabolism, Graduate School of Medicine, The University of Tokyo, 7-3-1, Hongo, Bunkyo-ku, Tokyo 113-0033, Japan; 2 Core Research for Evolutional Science and Technology (CREST), Japan Science and Technology Agency (JST), Chiyoda-ku, Tokyo 102-0076, Japan; 3 Evolutionary Morphology Laboratory, RIKEN, 2-2-3 Minatojimaminami-machi, Chuo-ku, Kobe 650-0047, Japan; 4 Division of Human Embryology, Department of Anatomy, Iwate Medical University, 2-1-1 Nishitokuta, Yahaba-cho, Shiwa-gun, Iwate 028-3694, Japan; 5 Évolution des Régulations Endocriniennes, CNRS UMR 7221, Muséum National, d'Histoire Naturelle, Paris, Cedex 05 75231, France; 6 Division of Cardiovascular Development and Differentiation, Department of Pediatric Cardiology, Medical Research Institute, Tokyo Women's Medical University, 8-1 Kawada-cho, Shinjuku-ku, Tokyo 162-8666, Japan; 7 Institute for Biology and Mathematics of Dynamical Cell Processes (iBMath), The University of Tokyo, 3-8-1 Komaba, Tokyo 153-8914, Japan

## Abstract

The amniote middle ear is a classical example of the evolutionary novelty. Although paleontological evidence supports the view that mammals and diapsids (modern reptiles and birds) independently acquired the middle ear after divergence from their common ancestor, the developmental bases of these transformations remain unknown. Here we show that lower-to-upper jaw transformation induced by inactivation of the Endothelin1-Dlx5/6 cascade involving *Goosecoid* results in loss of the tympanic membrane in mouse, but causes duplication of the tympanic membrane in chicken. Detailed anatomical analysis indicates that the relative positions of the primary jaw joint and first pharyngeal pouch led to the coupling of tympanic membrane formation with the lower jaw in mammals, but with the upper jaw in diapsids. We propose that differences in connection and release by various pharyngeal skeletal elements resulted in structural diversity, leading to the acquisition of the tympanic membrane in two distinct manners during amniote evolution.

## Abstract (web summary)

The evolution of the amniote middle ear remains unclear. Here, the authors show that inactivation of the Edn1-Dlx5/6 cascade during development results in loss of the tympanic membrane in mouse and duplication in chicken, which suggests independent evolution of the tympanic membrane in different amniotes.

## Graphical abstract

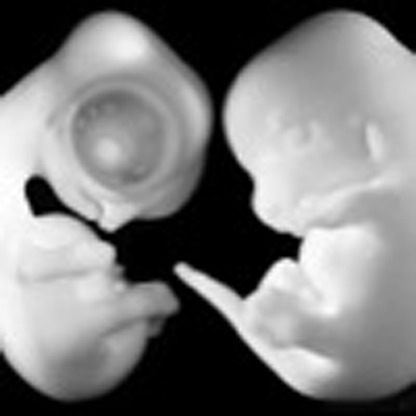

The evolution of an impedance-matching auditory system, in which sound is mechanically transferred to the cochlea via the tympanic membrane (TM) and the middle ear ossicle(s), was a key adaptation to terrestrial life for vertebrates^1^. Crown synapsids (modern mammals) possess three middle ear ossicles, the malleus, the incus and the stapes. The malleus and incus, which are respectively homologous with the articular and quadrate, form the primary jaw joint (PJJ) between the upper and lower jaws, and are mostly derived from the first pharyngeal arch (PA1), whereas the stapes is derived from the second pharyngeal arch (PA2)^2^,^3^,^4^. In contrast, diapsids (modern reptiles and birds) possess only a single ossicle, the columella auris, which is homologous with parts of the mammalian stapes. Paleontological observation indicates that the hyomandibular of basal amniotes braced between the braincase and the jaw, and did not connect to the TM as seen in crown diapsids^5^,^6^; their phylogenetic relationship suggests that mammals, diapsids and probably extinct parareptiles independently acquired the middle ear^7^,^8^,^9^. However, the developmental bases for these different middle ear patterns have remained elusive.

One difficulty in characterizing the distinct middle ears between mammals and diapsids arises from the fact that morphological homology has been established for every skeletal and muscular element^2^,^10^,^11^. Curiously, however, in light of the surrounding soft-tissue anatomy, significant differences have been identified in the relative positions of the TM in these groups^2^,^3^. In diapsids the TM attaches to the quadrate, an upper jaw element, while in mammals it spans the tympanic ring, an angular homologue associated with the lower jaw. This raises the possibility that despite the fact that they are derived from histologically comparable elements (endodermal and ectodermal invaginations and intervening tissues), the TMs were independently acquired in synapsids and diapsids, which exhibit distinct developmental topographies^2^,^3^. Consistent with this, non-identical positions of the external auditory meati (including the ectodermal component of the TM) have been reported in these lineages^12^,^13^. The key to understanding the distinct middle ear patterns may thus lie in the evolutionary relationship between TM-external auditory meatus and skeletal elements, making it crucial to dissect the developmental relations of these components during ontogeny.

In mammals, coupling between the TM and lower jaw components has been demonstrated by *Goosecoid* (*Gsc*) loss-of-function experiments^14^; however, the equivalent experiment has not been performed in chickens. We reasoned that if the evolutionary acquisition of the TM was coupled with patterning of the upper or lower jaw, experimental transformation of the upper/lower jaw should differentially affect the development of TMs between synapsids and diapsids. Endothelin1 (Edn1) signalling regulates *Dlx5/6* in the ventral region of the pharyngeal arches to specify the lower jaw and the PJJ by establishing the PA *Dlx*-code^15^,^16^,^17^. Inactivation of Edn1/Endothelin receptor type-A (Ednra) signalling in the mouse results in loss of lower jaw identity and transformation into a mirror-image duplication of the upper jaw, a phenotype closely resembling that of *Dlx5/6*-null mice^15^,^18^,^19^,^20^,^21^. Conversely, ectopic expression of *Edn1*, which in turn induces ectopic *Dlx5/6* in dorsal PAs, results in duplication of the lower jaw, including the tympanic ring^22^. Thus, experimental manipulation of Edn1 signalling in PAs may be a suitable approach to the study of TM formation.

Here we show that lower-to-upper jaw transformation induced by inactivation of the Edn1-Dlx5/6 cascade involving *Gsc* results in loss of the TM in mouse, but causes duplication of the TM in chicken, indicating that the TMs of mammals and diapsids are coupled with the development of the lower and upper jaws, respectively. Furthermore, in contrast to the classical hypothesis that the ventral swelling of the first pharyngeal pouch (PP1) enables acquisition of the lower-jaw-associated TM in mammals^23^, our comparative anatomical analysis indicates that the dorsal shift of the PJJ towards PP1 leads to differential coupling of the TM to jaw skeletons in avians and mammals. This report endows developmental and molecular evidence to support independent origin of the middle ear in different amniote taxa and the novel insight into the developmental process that led to these distinct middle ear patterns.

## Results

### Independent developmental origin of the TM in mammals and diapsids

**Figure 1:**
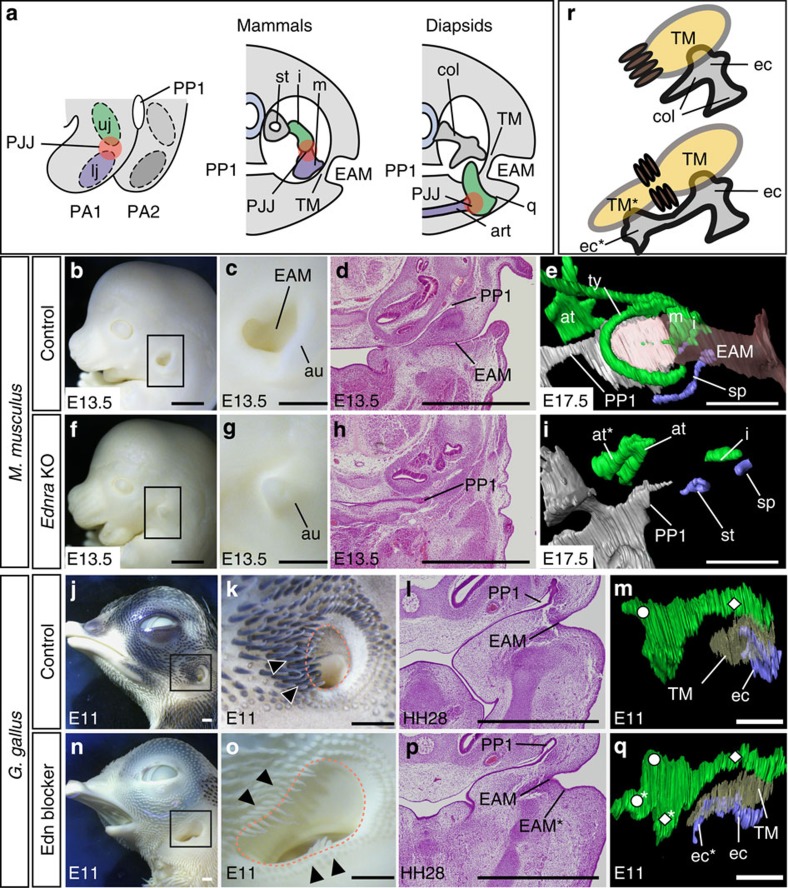
Comparison of middle ear phenotypes induced by inhibition of the Edn1-Dlx5/6 cascade in mouse and chicken. (**a**) Development and morphology of amniote middle ears. Generalized scheme of the first (PA1) and second (PA2) pharyngeal arches (left), transverse section of the middle ear in mammals (middle) and diapsids (right). Three ear ossicles, malleus (m), incus (i) and stapes (st) are present in mammals, while diapsids have only a single ossicle, columella auris (col). The TM comprises the first pharyngeal pouch (PP1) and external auditory meatus (EAM). Left lateral appearance (**b**,**c**,**f**,**g**), horizontal sections (**d**,**h**) and ventrolateral views of the three-dimensional (3D) reconstructed middle ear (**e**,**i**) in control (**b**–**e**) and *Ednra*-null (**f**–**i**) mice. Higher magnification of boxes in **b**,**f** are shown in **c**,**g**, respectively. Skeletal components derived from PA1 (green) and PA2 (blue), EAM (pink) and PP1 (grey) are shown in **e**,**i**. Left lateral appearance (**j**,**k**,**n**,**o**), horizontal sections (**l**,**p**) and ventrolateral views of the 3D reconstructed middle ear (**m**,**q**) in control (**j**–**m**) and Edn-blocker-treated (**n**–**q**) chickens. (**k**,**o**) Higher magnifications of **j**,**n**, respectively. Outer edges of EAM is delineated by red dashed lines and ventral auricular feathers are indicated by arrowheads (**k**,**o**); they delineate the ventral margin of the EAM in control (**k**) and the supernumerary EAM was formed ventrally to them (**o**). Note that pigmentation is also impaired by Edn blockage, which acts on the Ednrb receptor in melanocytes^51^ (**n**,**o**). White circles and lozenges indicate the pterygoid and otic process of the quadrate, respectively, and TM is coloured translucent yellow (**m**,**q**). The extracolumella (a lateral part of the columella auris that attaches to the TM in control) is duplicated and attached to the ectopically formed TM in Edn-blocker-treated chicken embryos (**m**,**q**,**r**; also see Supplementary Fig. 1j,n). (**r**) Summary of the induced middle ear phenotypes in chicken embryos. Dark brown ovals indicate the ventral auricular feathers. art, articular; at, ala temporalis; au, auricle; ec, extracolumella; lj, lower jaw; PJJ, primary jaw joint; q, quadrate; sp, styloid process; ty, tympanic ring; uj, upper jaw; *duplicated elements. Scale bars, 500 μm for **c**,**g**, 1 mm for other panels.

To dissect developmental relationships between TM-external auditory meatus and jaw skeletal elements, we first analysed *Ednra*-null mice; in these animals, invagination of the external auditory meatus was absent, with loss of the TM (Fig. 1f–i; compare with Fig. 1b–e). The TM of therian mammals consists of two regions, namely the pars tensa and pars flaccida, and some classical studies suggested the homology between the latter and the reptilian TM^23^. We confirmed, however, the entire TM was lost in the *Ednra*-null mouse. These observations indicate that formation of the TM in the mouse is coupled with development of the lower jaw.

We next inhibited the Edn1-Dlx5/6 cascade in chicken embryos using bosentan, an Edn receptor antagonist. This resulted in hypoplasia of lower jaw skeletons (Fig. 1j,n and Supplementary Fig. 1g,k), downregulation of *Dlx5/6* (Supplementary Fig. 1a,b,d,e) and transformation of the lower jaw morphology, mimicking upper jaw identity (Supplementary Fig. 1g–n) as in the case of the mouse^18^,^19^,^20^. Namely, lower jaw skeletons such as the articular, dentary, angular and the Meckel's cartilage were disturbed, and instead ectopic upper jaw-like skeletons that, because of their relations, correspond to what in the adult we would homologize with the maxilla, jugal, palatine, pterygoid, and otic and pterygoid processes of the quadrate appeared in the lower jaw position in a mirror-image pattern (Supplementary Fig. 1g–n).

In contrast to the mouse, however, a supernumerary external auditory meatus appeared ventral to the original one (Fig. 1j–l,n–p), resulting in duplication of the TM (Fig. 1m,q,r and Supplementary Fig. 1o–r). The supernumerary TM was associated with the duplicated extracolumella (a part of the columella auris attached to the TM) and formed ventral to the typical long feathers that delineate the ventral margin of the control TM (Fig. 1k,m,o,q,r). Thus, although perturbation of the same developmental signalling pathway resulted in similar jaw skeletal patterning phenotypes in mouse and chicken embryos, the resulting TM phenotypes were different.

**Figure 2:**
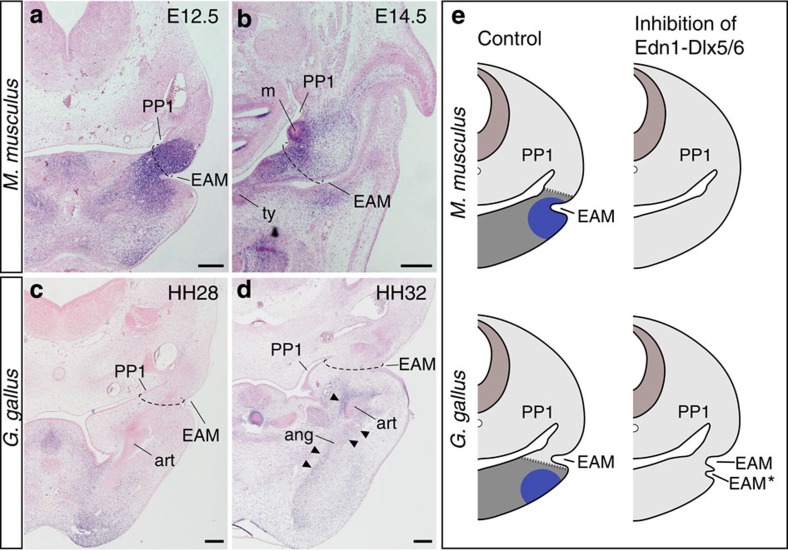
Expression pattern of *Gsc* in mouse and chicken. (**a**–**d**) *Gsc* expression in transverse sections of the mouse embryonic day 12.5 (**a**) and 14.5 (**b**), and chicken stage 28 (**c**) and 32 (**d**) embryos. The expression signals along the forming articular (art) and angular (ang) in **d** are indicated by arrowheads. Dashed lines indicate the planes of future TM. (**e**) The jaw regionalization and the TM formation. The lower jaw region (grey) and expression of *Gsc* (blue) are shown. EAM, external auditory meatus; m, malleus; PP1, first pharyngeal pouch. Scale bars, 200 μm.

To understand why inhibition of the Edn1-Dlx5/6 cascade induced distinct TM phenotypes, we investigated gene expression patterns involved in jaw and middle ear patterning in both species. *Gsc* is a downstream target of the Edn1-Dlx5/6 cascade in both mouse^24^,^25^ and chicken (Supplementary Fig. 1c,f). In the mouse, *Gsc* inactivation results in loss of the external auditory meatus and the tympanic ring (homologue of the angular in diapsids)^14^, suggesting that the external auditory meatus is induced as a lower jaw component. Indeed, in mice *Gsc* expression was associated with the TM and tympanic ring primordia (Fig. 2a,b). In contrast, chicken *Gsc*, although also detected in the angular, was not found in the proximity of the forming TM, which is located more dorsally (Fig. 2c,d). The latter expression pattern is also found in gecko (a lepidosaur; see Supplementary Fig. 2), indicating that this expression pattern is conserved in diapsids. These results suggest that although *Gsc* is expressed in the proximal part of the ventral PA1 in both mammals and diapsids, it plays a role in TM patterning only in mammals. Thus, although the TM of mammals and diapsids are functionally comparable, they are coupled with distinct PA1 components (Fig. 2e).

### The relative positions of the PJJ and PP1 led to the distinct TM formation

**Figure 3:**
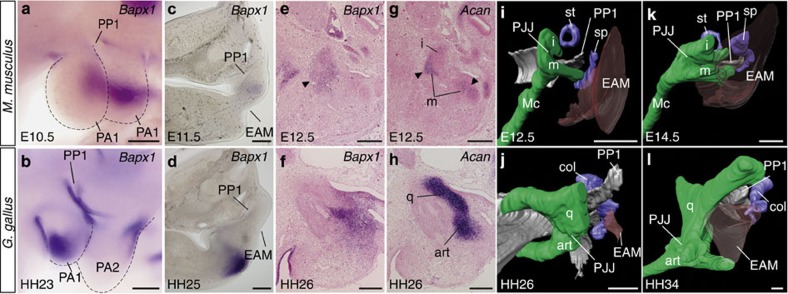
Comparison of development of the TM and PJJ in mouse and chicken. (**a**–**d**) *Bapx1* expression in pharyngeal arches of the embryonic day 10.5 mouse (**a**), stage 23 chicken (**b**) in lateral view and transverse sections of the embryonic day 11.5 mouse (**c**) and stage 25 chicken (**d**). *Bapx1* and *Aggrecan* (*Acan*) expression in sagittal serial sections of embryonic day 12.5 mouse (**e**,**g**) and stage 26 chicken (**f**,**h**). The expression signals in the incus (i) and malleus (m) are indicated by arrowheads (**e**,**g**). (**i**–**l**) Three-dimensional reconstruction of forming the pharyngeal skeleton and TM of embryonic day 12.5 (**i**) and 14.5 (**k**) mouse, and stage 26 (**j**) and 34 (**l**) chicken (coloured same as in Fig. 1). art, articular; col, columella auris; EAM, external auditory meatus; Mc, Meckel's cartilage; PA1–2, pharyngeal arches 1–2; PJJ, primary jaw joint; PP1, first pharyngeal pouch; q, quadrate; sp, styloid process; st, stapes. Scale bars, 200 μm.

The different roles of *Gsc* in mouse and chicken embryos appear to reflect differences in the relative topographical relationships between the TM (reflecting the position of PP1) and the PJJ in these species. In embryos of both species, PP1 forms in the same relative position between PA1 and PA2 (Supplementary Fig. 3). We examined expression of *Bapx1*, a marker gene for PJJ mesenchyme^26^,^27^, and found that in the mouse embryo it was expressed in proximity of the PP1 more dorsally than in the chicken embryo (Fig. 3a–d). This difference became more pronounced during development, resulting in the formation of the procartilaginous PJJ adjacent to PP1 in mouse and ventral to PP1 in chicken (Fig. 3e–j); the external auditory meatus invaginates ventral to the PJJ in mouse and dorsal to the PJJ in chicken (Fig. 3i–l). Notably, dorsoventral positional relationships between the external auditory meatus and PP1 did not show significant differences; the external auditory meati invaginate slightly ventral to PP1 in both species (Fig. 3i–l). Thus, the relative positions of skeletal and TM components differ in mammals and diapsids.

To explain the inconsistent positioning of TMs in mammals and diapsids, classical hypotheses assumed either a ventral shift of the ancestral TM^28^,^29^ or a *de novo* acquisition of the TM, associated with a hypothetical ventral swelling of the middle ear cavity in mammals^23^. The latter hypothesis is inconsistent with the mammal-specific distribution of neural crest cell-derived epithelium in the middle ear cavity^30^. We showed that both TMs differentiate from PP1, an embryonic anlage developmentally equivalent in mouse and chicken, and that there is no secondary ventral swelling of PP1 in the mouse (Fig. 3i,k). Rather, PJJ primordia develop in different positions relatively to PP1 in avian and mammalian embryos (Fig. 3). The most plausible explanation would simply be that a topographical frameshift between PP1 and the PJJ developmentally leads to different TMs and middle ears in mammals and diapsids.

## Discussion

Morphological evolution of the mammalian middle ear has long remained a conundrum^2^,^3^,^4^. To provide a developmental basis for the recent paleontological hypothesis that mammals and diapsids independently acquired the middle ear^5^,^6^,^7^,^8^, we analysed the dorsoventral patterning of TMs through a developmental biological approach in mouse and chicken embryos. Given that inactivation of the Edn1-Dlx5/6 cascade resulted in comparable skeletal phenotypes^18^,^19^,^20^ (Supplementary Fig. 1), and *Bapx1* and *Gsc* are expressed in a homologous set of skeletal elements in these animals (Figs 2 and 3), it appears that the gene regulatory networks of PAs are highly conserved, to assure the morphological homologies of PA skeletal elements in amniotes^17^. However, the TMs of mammals and diapsids are topologically distinct, although both are generated through interactions among PP1, the external auditory meatus and the surrounding mesenchyme^31^, and early PP1 is homologous in these animals (Fig. 3 and Supplementary Fig. 3). These findings collectively indicate that the difference in developmental mechanisms for these TMs should not be regarded as caused by a simple developmental system drift^32^,^33^, but rather reflects unshared evolutionary events underlying their acquisition. We thus propose that the developmental programmes for skeletal specification and TM formation are decoupled primarily, and that TM formation was secondarily coupled with lower and upper PA1 components in mammals and diapsids, respectively.

**Figure 4:**
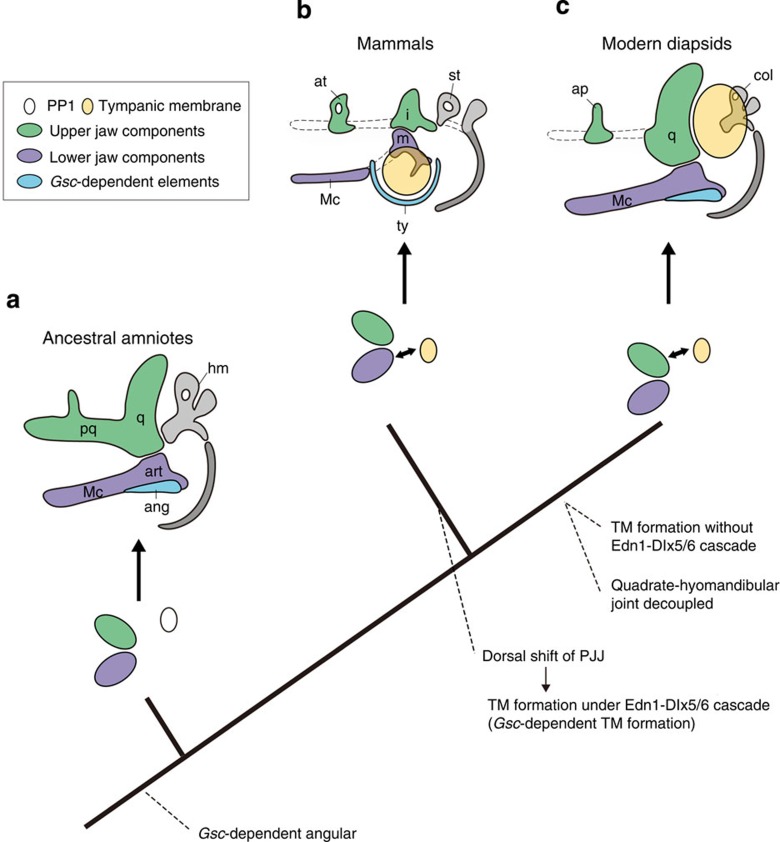
Hypothesized scenario of the TM and middle ear evolution in amniotes. (**a**) In ancestral amniotes, the position of PJJ is thought to have been distant from PP1. The TM had not yet been obtained. (**b**) In mammalian ancestors, the PJJ shifted dorsally to the proximity of PP1, leading to the coupling of the TM and lower jaw specifications to form the Edn1-Dlx5/6-dependent TM in the lower jaw domain. This TM spans the angular, dependent on *Gsc* expression. (**c**) In modern diapsids, the position of the PJJ retains the ancestral state. The hyomandibular has been decoupled from the quadrate and established a connection with the Edn1-Dlx5/6-independent TM in the upper jaw domain. ang, angular; art, articular; ap, ascending process; at, ala temporalis; col, columella auris; hm, hyomandibular; i, incus; m, malleus; Mc, Meckel's cartilage; PP1, first pharyngeal pouch; pq, palatoquadrate; q, quadrate; st, stapes; ty, tympanic ring.

The above noted developmental mechanism of the middle ear has a shallower history than the split of the mammalian and diapsid lineages, which occurred 50 million years after vertebrate terrestrialization^34^,^35^. Developmental analyses in outgroups of amniotes (axolotl and shark; Supplementary Fig. 4) and the similarity of adult morphology to basal amniotes^5^,^36^,^37^,^38^ suggest that the positional interrelationship between PP1 and PJJ in crown diapsids represents the plesiomorphic state for amniotes (Fig. 4a). Stem synapsids and stem diapsids exhibited jaw suspension using the hyomandibular articulated with the quadrate, which later became relaxed due to the rearrangement of bony connections in the skull of the diapsid lineage, and ultimately loss of the quadrate-hyomandibular articulation^39^. In the synapsid lineage, however, the original articulation among the hyomandibular, quadrate and articular was retained, disabling the possession of hyomandibular-associated TM^7^. The dorsal positional shift of the PJJ in embryonic development would have permitted the formation of the unique lower jaw-associated TM in mammalian ancestors. Thus, differences in the connection and release among these skeletal elements may have served as a key innovation, marking this watershed in the subsequent evolution of two different origins of middle ears and TMs during amniote evolution as a typical case of convergence (Fig. 4b,c). It is fascinating to see how such functionally and histologically similar structures can arise during evolution, based not only on shifted sets of embryonic anlagen, but on non-equivalent developmental programmes as well.

## Methods

### Sample collection

Mouse *Mus musculus* embryos were collected and staged according to Theiler^40^, considering the noon of the day on which vaginal plugs were detected after timed mating as E0.5, from 8- to 40-week-old females. *Ednra*-null (*Ednra*^*GFP*^) locus was generated by insertion of an *EGFP* cassette into the *Ednra* locus, using Cre recombinase-mediated cassette exchange system on *Ednra*^*neo/+*^ embryonic stem cells, which contain exchangeable floxed site in the *Ednra* locus^41^. Mice were maintained on a mixed C57BL/6J × ICR background and housed in an environmentally controlled room at 23±2 °C, with a relative humidity of 50%–60% and under a 12-h light:12-h dark cycle. Fertilized eggs of chicken *Gallus gallus* were incubated in a humidified atmosphere at 37 °C –38 °C and staged according to Hamburger and Hamilton^42^. Embryos of axolotl *Ambystoma mexicanum*, catshark *Scyliorhinus torazame* and gecko *Paroedura picta* (formerly *P. pictus*) were collected and staged according to previous studies^43^,^44^,^45^. All animals were analysed without distinction of sex. All animal experiments were performed in accordance with the institutional guidelines of the Animal Care and Use Committees of the University of Tokyo, RIKEN CDB and the Iwate Medical University.

### Molecular cloning

Total RNA was extracted from a whole embryo of *P. picta*, using TRIZOL reagent (Life Technologies). Degenerate reverse transcriptase–PCR and rapid amplification of complementary DNA ends PCR were performed to amplify fragments of *Gsc* using the GeneRacer Kit (Life Technologies). These fragments were sequenced using an ABI 3130XL automated sequencer (Applied Biosystems). A putative amino acid sequence of the clone was aligned with *Gsc* family genes in representative species. The molecular phylogenetic tree was inferred using the neighbour-joining method^46^.

### Pharmacological inactivation of Edn signalling *in ovo*

The method has been described previously^47^,^48^. Bosentan (Actelion, Ltd) was suspended in the corn oil (Sigma) in 37 °C water bath for 30 min at the 5 mg ml^−1^. A small window was opened in the shell of 48-h-incubated eggs and 30 μl bosentan-diluted oil drop was introduced onto the shell membrane, while only oil was given to the control group. The shell opening was sealed with tape and eggs were incubated again until dissection.

### Skeletal staining

Skeletal staining of embryos with alizarin red/alcian blue staining was performed according to previously described protocols^49^, with slight modifications. Samples were fixed in 95% ethanol for 1 week, placed in acetone for 2 days and incubated with 0.015% alcian blue 8GS, 0.005% alizarin red S and 5% acetic acid in 70% ethanol for 3 days. After washing in distilled water, the samples were cleared in 1% KOH for several days and in 1% KOH glycerol series until the surrounding tissues turned transparent. The specimens were stored in glycerol.

### *In situ* hybridization

Digoxigenin (DIG)-labelled riboprobes for mouse *Bapx1*, *Pax1*, *Aggrecan*, chicken *Dlx5*, *Dlx6*, *Bapx1*, *Gsc*, shark *Bapx1* and gecko *Gsc* were generated by DIG RNA labelling kit (Roche) based on the nucleotide sequences NM_007524, NM_008780, L07049, NM_204159, AY_640308, NM_204137, NM_205331, AB293587 and AB985798 deposited in GenBank, respectively. The nucleotide sequence of mouse *Gsc*, chicken *Aggrecan*, and quail *Pax1* were gifts from Gen Yamada (Kumamoto University), Atsushi Kuroiwa (Nagoya University) and Hirohiko Aoyama (Hiroshima University), respectively. Embryos were fixed with Serra's fixative or 4% paraformaldehyde, dehydrated, embedded in paraffin and sliced into 6–10 μm sections. *In situ* hybridization for the sections were performed by a Ventana Discovery XT system, using a BlueMap NBT/BCIP substrate kit and a Red counterstain II (Roche-Ventana Medical Systems). Whole-mount *in situ* hybridization was performed as previously described^50^, with slight modifications. Embryos were fixed with 4% paraformaldehyde, dehydrated and treated with hydrogen peroxide solution in methanol (1:5) overnight. The specimens were rehydrated in PBT (PBS containing 0.1% Tween 20), digested with 20 μg ml^−1^ proteinase K (Roche) and refixed with 4% PFA/PBT containing 0.2% glutaraldehyde. The samples were incubated in hybridization buffer with 0.1–1 μg ml^−1^ DIG-labelled riboprobe for 24 h at 65 °C. After hybridization, the specimens were incubated with 50 μg ml^−1^ RNaseA for 30 min at room temperature, washed with 2 × SSC and 0.2 × SSC containing 0.3% CHAPS at 65 °C. The embryos were blocked with 1.5% blocking reagent (Roche) and incubated with alkaline phosphatase-conjugated anti-DIG Fab fragments (diluted 1:4,000; Roche) at 4 °C overnight. The specimens were washed 12 times with Maleic acid buffer (0.15 M Maleic acid, 0.15 M NaCl, 0.1% Tween 20) at room temperature. Alkaline phosphatase activity was detected with NBT/BCIP (Roche). Following *in situ* hybridization, the whole-mount samples hybridized with *Bapx1* riboprobe were embedded in gelatin and sections (120 μm) were generated with a vibrating microtome.

### Histological analysis and 3D reconstruction

Each paraffin-embedded section (6–12 μm) was stained by haematoxylin and eosin. For the stage 34 chicken and embryonic day 14.5 mouse embryos, histological sections were stained with 0.1% alcian blue after haematoxylin and eosin staining. Procartilaginous development in histological sections of the stage 26 chicken and embryonic day 12.5 mouse were detected by *in situ* hybridization with *Aggrecan* riboprobe. For three-dimensional reconstruction, digital images of the stained sections were loaded into Amira (Visage Imaging, Inc.) with a voxel size appropriate to section thickness.

## Author contributions

T.K., M.T., S.K. and H. Kur. designed the study. T.K., H. Kum., K.M., Y.K. and S.M.-T. performed experiments for bosentan-treated chicken embryos. T.K., N.N., H. Kum. and G.L. collected data from *Ednra*-null mouse embryos. M.T., T. Hirai., J.H. and S.K. collected data from wild-type mouse and chicken embryos. T. Hirasawa., T. Hirai. and M.T. performed the gecko analysis. N.A. and M.T. collected data, using shark embryos. M.T. obtained data from axolotl embryos. T.K., M.T., T. Hirasawa, G.L., H. Kur. and S.K. wrote the manuscript. All of the authors discussed the results and commented on the manuscript.

## Additional information

**Accession codes:** The Gecko *Gsc* sequences generated in this study have been deposited in DNA Data Bank Japan (DDBJ) under accession code AB985798.

**How to cite this article:** Kitazawa, T. *et al*. Developmental genetic bases behind the independent origin of the tympanic membrane in mammals and diapsids. *Nat. Commun.* 6:6853 doi: 10.1038/ncomms7853 (2015).

## Supplementary Material

Supplementary Information Supplementary Figures 1-4 and Supplementary References
