# Supplementary material for: Developmental genetic bases behind the independent origin of the tympanic membrane in mammals and diapsids
Source: Nat Commun. 2015 Apr 22;6:6853. doi: 10.1038/ncomms7853 (PMC4423235; doi:10.1038/ncomms7853)
Supplement: Supplementary Information — Supplementary Figures 1-4 and Supplementary References [file ncomms7853-s1.pdf]

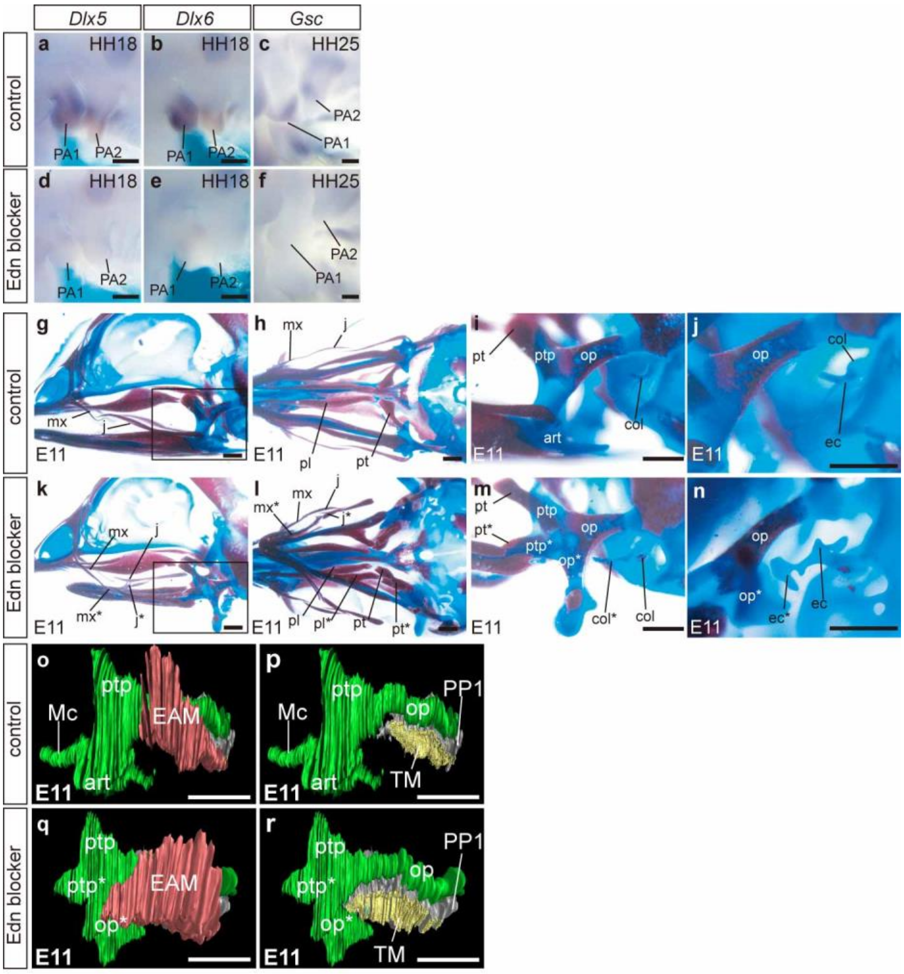

Supplementary Figure 1| Treatment of Edn-blocker in chickens induces homeotic transformation of the lower jaw into the upper jaw, and duplication of the tympanic membrane. (**a-f**) *In situ* hybridization of *Dlx5* (**a,d**), *Dlx6* (**b,e**) and *Gsc* (**c,f**) riboprobes in the pharyngeal arches of stage 18 (**a,b,d,e**) and 25 (**c,f**) control (**a-c**) and Edn-blocker treated (**d-f**) chicken embryos. (**g-n**) Skeletal staining of lateral views (**g,i,k,m**), ventral views (**h,l**) and ventrolateral views (**j,n**) of control (**g-j**) and Edn-blocker treated (**k-n**) chicken. **i** and **m** are higher magnification of boxes in **g** and **k**, respectively. (**o-r**) Left lateral views of the 3D reconstructed middle ear of embryonic day 11 control (**o,p**) and Edn-blocker treated (**q,r**) chicken embryos. Skeletal components derived from PA1 (green) and PA2 (blue), TM (yellow), EAM (pink) and PP1 (gray) are shown. EAM is removed in **p** and **r**. art, articular; col, columella auris; ec, extracolumella; EAM, external auditory meatus; j, jugal; mx, maxilla; op, otic process of quadrate; PA1-2, pharyngeal arches 1-2; PP1, first pharyngeal pouch; pl, palatine; pt, pterygoid; ptp, pterygoid process of quadrate; q, quadrate; TM, tympanic membrane; *, duplicated elements. Scale bars, 200 µm (**a-f**), 1 mm (**g-r**).

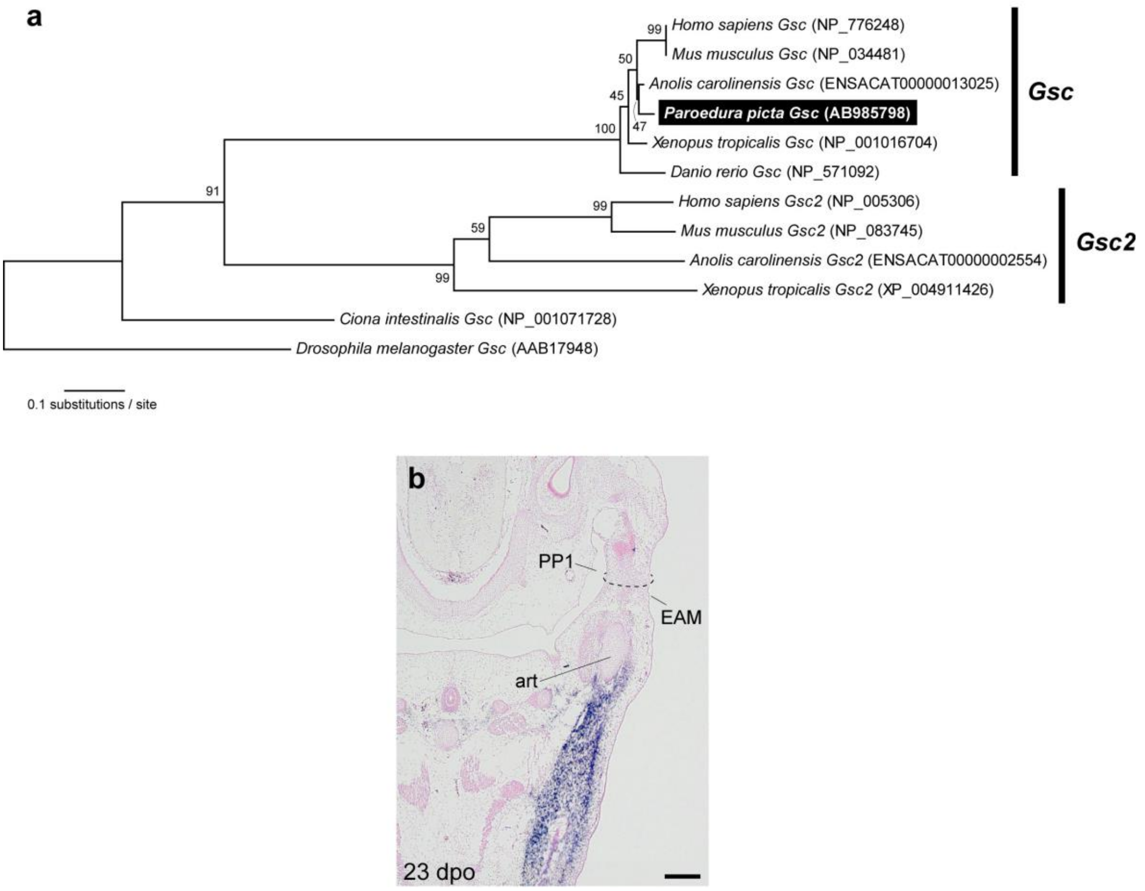

Supplementary Figure 2| Molecular phylogenetic and expression analysis of gecko *Gsc*. (**a**) Neighbor-joining phylogenetic tree showing the relationship of *Gsc* and *Gsc2* genes in gecko *Paroedura picta* and representative species. Bootstrap values (with 1,000 replications) are shown on the nodes. *Gsc2* is unlikely to be involved in middle ear formation because this gene is not expressed in the pharyngeal arches during development[1,2]. (**b**) Expression pattern of *Gsc* in a transverse section of *P. picta* 23 days post oviposition (dpo) embryo. Dashed line indicates the plane of future TM. art, articular; EAM, external auditory meatus; PP1, first pharyngeal pouch. Scale bar, 200 μm.

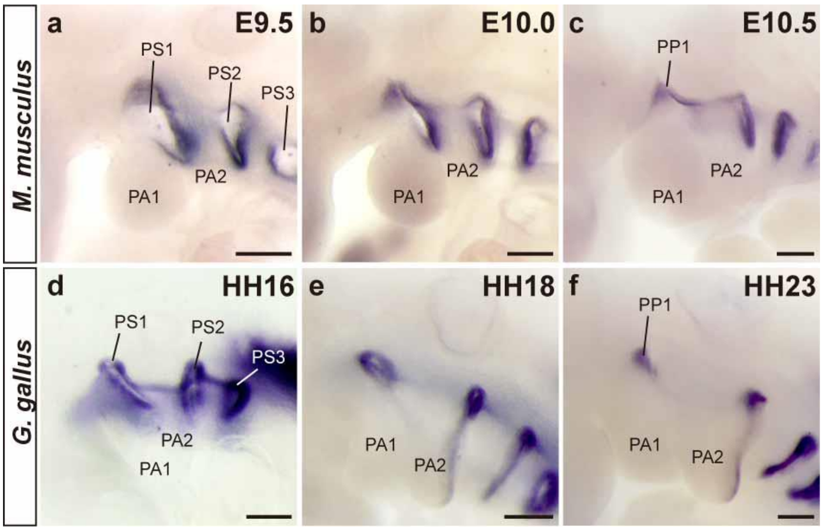

Supplementary Figure 3| Development of the first pharyngeal pouch in the mouse and chicken. Expression pattern of *Pax1,* a molecular marker for the pharyngeal endoderm, in the pharyngeal slits of mouse embryonic day 9.5 (**a**), 10.0 (**b**) and 10.5 (**c**), and chicken stage 16 (**d**), 18 (**e**) and 23 (**f**) embryos in lateral view. PP1 forms by the same manner in almost the same position during pharyngula stage; it forms by the closure of the first pharyngeal slit from ventral to dorsal both in mouse and chicken embryos. PA1-2, pharyngeal arches 1-3; PP1, first pharyngeal pouch; PS1-3, pharyngeal slits 1-3. Scale bars, 200 μm.

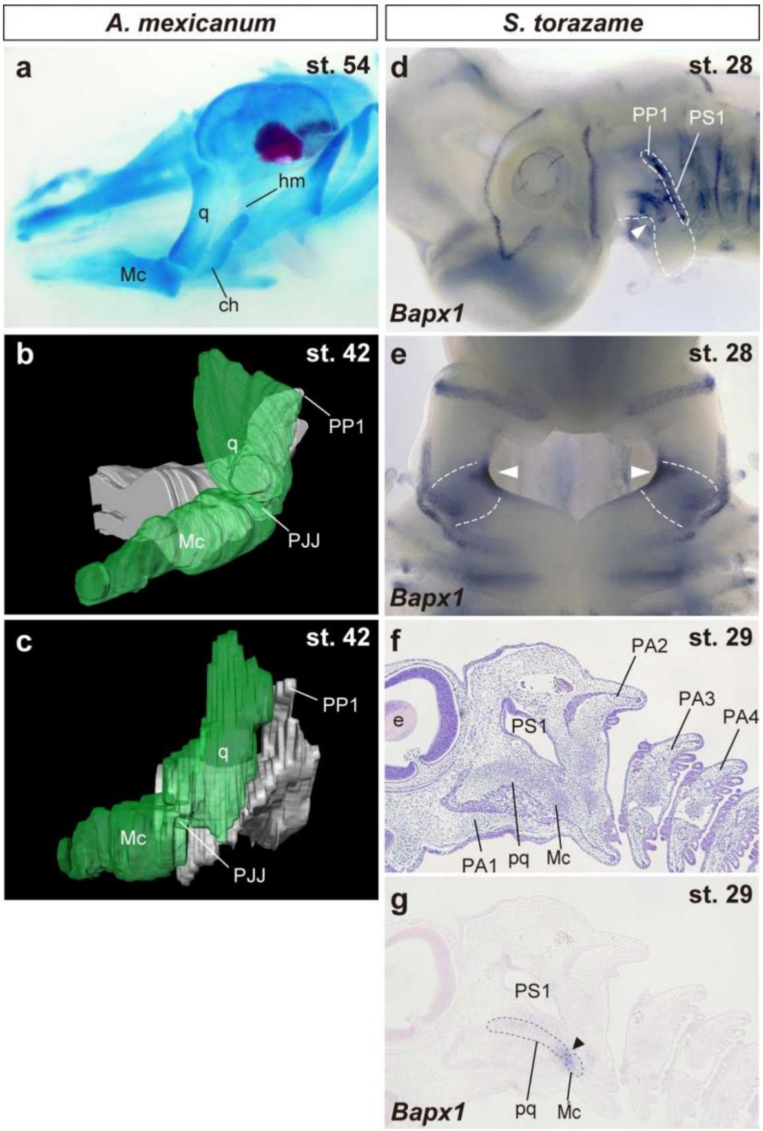

Supplementary Figure 4| Relative positional relationships in forming skeletal elements and first pharyngeal pouch in the axolotl and shark. Development of PA1 pharyngeal skeletons in axolotl, *Ambystoma mexicanum* (**a-c**) and shark, *Scyliorhinus torazame* (**d-g**). (**a**) Alcian blue staining of the axolotl embryo (st. 54). (**b,c**) dorsolateral (**b**) and lateral (**c**) view of the 3D reconstractions of the quadrate, Meckel's cartilage and pharyngeal endoderm in st. 42 embryo clearly show that the PJJ is distant from PP1. (**d-g**) *Bapx1* expresion in lateral (**d**), ventral (**e**) view of whole-mount sample and sagittal section (**g**) in st. 28 and 29 shark embryos. (**f**) shows H-E staining of a section corresponding to (**g**). *Bapx1* expression in mesenchyme (surrounded by dashed line in **e**) is detected around the maxillary-mandibular constriction (white arowhead in **d** and **e**), where the PJJ is forming (black arrowhead in **g**). In the shark, PP1 can be defined as the

dorsalmost area of the first pharyngeal slit (**d**), because this area is ventrally adjacent to the geniculate ganglion of the facial nerve[3]. The PJJ is clearly distant from PP1 in the shark embryo (**d**). ch, ceratohyal; hm, hyomandibular; Mc, Meckel's cartilage; PA1-4, pharyngeal arches 1-4; PJJ, primary jaw joint; PP1, first phayrngeal pouch; pq, palatoquadrate; PS1, first pharyngeal slit; q, quadrate. Scale bars, 500 μm (**a,d-g**), 100 μm (**b,c**).

Supplementary References

1. Gottlieb S, Hanes SD, Golden JA, Oakey RJ, Budarf ML. Goosecoid-like, a gene deleted in DiGeorge and velocardiofacial syndromes, recognizes DNA with a bicoid-like specificity and is expressed in the developing mouse brain. *Hum. Mol. Genet.* **7**, 1497-1505 (1998).

2. Funato H, et al. Loss of Goosecoid-like and DiGeorge syndrome critical region 14 in interpeduncular nucleus results in altered regulation of rapid eye movement sleep. *Proc. Natl. Acad. Sci. U S A* ***107***, 18155-18160 (2010).

3. O'Neill, P., McCole, R. B., Baker, C. V. A molecular analysis of neurogenic placode and cranial sensory ganglion development in the shark, Scyliorhinus canicula. *Dev. Biol.* **304**, 156-181 (2007).
